# Alcohol-related harm among university students in Hanoi, Vietnam

**DOI:** 10.3402/gha.v6i0.18857

**Published:** 2013-02-01

**Authors:** Pham Bich Diep, Ronald A. Knibbe, Kim Bao Giang, Nanne De Vries

**Affiliations:** 1 Institute of training of Preventive Medicine and Public Health, Hanoi Medical University, Hanoi, Vietnam; 2 Department of Health Promotion, CAPHRI School for Public Health and Primary Care, Maastricht University, Maastricht, The Netherlands

**Keywords:** female students, male students, alcohol-related harm, type of harm, drinking patterns, Vietnam

## Abstract

**Introduction and Aim:**

This study examines the prevalence of and risk factors for alcohol-related harm and types of harm among medical students from Hanoi Medical University (Vietnam). Risk factors include aspects of drinking patterns and relevant socio-demographic variables.

**Study Design and Methods:**

A cross-sectional study involving 1st to 6th year students (*N*=1216; response rate 96.5%). Of these, 210 students from each academic year were randomly selected from a sampling frame covering all students from each academic year. Data were collected using a questionnaire distributed in class by researchers. Drinkers completed 23 questions on alcohol-related harm categorized into: 1) ‘negative influence on daily activities’; 2) ‘social conflict’; 3) ‘loss of control, acute consequences, and withdrawal’; 4) ‘mental health conditions’; and 5) ‘physical and medical health problems’. Logistic and Poisson regression models were used to identify the predictors of alcohol-related harm and the amount of harm, respectively.

**Results:**

The prevalence of alcohol use associated with at least one or more of the five types of harm was higher in men (81.8%) than in women (60.4%). In female and male students, the most common harm category was ‘loss of control, acute consequences, and withdrawal’ (51.8 and 75.6%, respectively), followed by ‘negative influence on daily activities’ (29.4 and 55.8%, respectively). Age, living away from home, and average number of standard drinks per occasion among male drinkers, and age and frequency of drinking per week among female drinkers were associated with alcohol-related harm.

**Conclusions:**

These data suggest that alcohol-related harm represents a serious public health problem among young educated individuals in Vietnam. The risk factors indicate that prevention should be aimed at aspects of drinking patterns and specific subpopulations defined by gender, age, and (for men only) type of living situation.

Alcohol is the third leading cause of burden of disease worldwide. It is reported that out of all deaths among young people aged 15–29 years, 9% is related to alcohol (1). Harmful drinking patterns, such as binge drinking (mostly defined as drinking ≥6 glasses on one occasion), have increased among young adults and adolescents (2). Harmful drinking is also popular among college students (3, 4). A study among Vietnamese medical students indicated that the prevalence of alcohol consumption (65.5%) and alcohol-related problems (12.5%) is relatively high (5). However, data on the negative consequences of harmful drinking in this population are scarce.

Regarding alcohol-related harm, the prevalence of negative consequences of alcohol use is high in both male and female students in developed countries such as New Zealand and the USA (4, 6). After drinking, male and female students often experience blackouts, unintended/unprotected sexual activity, academic impairment, short and long-term physical illness, and poor mental health, as well as anti-social risk behavior, fights, and interpersonal violence (7–11). A study among young Australian students suggested that alcohol-related harm has increased dramatically in recent years (12); however, studies on alcohol-related harm among students in developing countries are still scarce. A study in Thailand also indicated that the prevalence of hangover, nausea, and vomiting among adolescent drinkers is high (46.9%) (13); however, information on alcohol-related harm among students in Vietnam is lacking. Two studies among adolescents/young adults in Vietnam examined the association between alcohol use and sexual behavior only; the results show a strong link between alcohol consumption and engaging in sexual behavior among both males and females (14, 15).

**Policy recommendations**

The findings from this study indicate that it is a particular concern to develop alcohol policies to reduce harmful use of alcohol among students. The following points summarize the key policy recommendations:

- More attention should be paid to reduce the harmful use of alcohol among students.
- Intervention should aim at risk factors, including aspects of drinking patterns and specific subpopulations defined by gender, age and (for men only) type of living situation.
- Education should be emphasized aiming at increasing awareness of students about the harmful use of alcohol.

Drinking patterns, in addition to quantity, are strongly related to harm. The volume of consumption (mostly expressed as the number of standard drinks per week or month) is an important determinant of alcohol-related harm (16, 17). Nevertheless, the same volume can still conceal very different drinking patterns. The aspect of drinking patterns most widely examined is that of binge drinking. Other measures for incidental high consumption, such as the greatest number of glasses consumed on one occasion and frequency of drunkenness, are also related to an increased risk for harm. A national survey of students at 140 campuses in the USA showed that frequent binge drinkers and infrequent binge drinkers were 25 and 5 times, respectively, more likely to have experienced at least five harms compared to non-binge drinkers (3). Notably, students who drink both heavily and frequently experienced negative consequences almost three times as often as those who drink heavily but less often (18).

Socio-demographic factors (such as age and gender) are also associated with alcohol-related harm. A study in the United Kingdom showed that older adolescents are more likely to report alcohol-related violence and alcohol-related regretted sex (19). Many studies in developed countries (e.g. USA, New Zealand, Australia, Sweden, and Germany) and in developing countries (e.g. Thailand and China) report a gender difference in drinking patterns that influence harms (13, 20–27). Similarly, a study among students in Vietnam reported that men were 14.3 times more likely to have alcohol problems compared with women (5).

The present study among Vietnamese students addresses three related questions: 1) What is the prevalence of alcohol-related harm in this group? 2) How are socio-demographic variables and drinking patterns associated with alcohol-related harm? 3) Do socio-demographic and drinking pattern variables explain the variation in the number of alcohol-related harms that students report?

## Methods

### Setting

A cross-sectional study was conducted between November 2008 and January 2009 at Hanoi Medical University (the oldest and largest medical university in Hanoi, Vietnam).

### Sample size and sampling

Within each academic year, the World Health Organization sample size calculation was applied to calculate a sample size, assuming a 45% prevalence of alcohol-related harms among drinkers (with a precision of ±0.2 and a 95% confidence level). Since we cannot select drinkers, the sample size required is calculated based on the prevalence of alcohol use among students, that is, 65% (5); therefore, a sample size of 180 students per academic year was needed. This number was increased by 5% to account for losses and by an additional 10% to control for confounding, yielding a sample size of 207 students for each academic year (rounded up to 210 students per academic year). The total sample size of 1260 students was also sufficient to achieve 90% power to detect an absolute difference of 15% in the proportions of having alcohol-related harm among male and female students (level of significance of 5%, and non-response rate and confounding control of 15%). Subsequently, 210 students per academic year were randomly selected from the register of medical students for each academic year (provided by the Department of Training and Education at Hanoi Medical University). At this stage, a total of 1260 university students from the 1st to 6th study years were selected. Finally, 1216 students (96.5%) participated in the study; 44 students (3.5%) declined to participate. The age and sex distribution of the non-respondents did not differ from that of the respondents.

### Data collection

A letter explaining the aims, assurance of confidentiality, and specification of the date, time, and place to fill in the questionnaire was delivered to the selected students by their class monitors. The investigators and research assistants were trained before data collection.

Each time point of data collection involved a maximum of 30 students, one investigator, and two research assistants. The structured questionnaire and pictures of the most common beverages in Vietnam (with their ethanol levels, and corresponding units for a standard drink) were distributed to the students by the research assistants. The investigators explained the definition of a standard drink (SD) used for this study (see ‘Measures’ below) and instructed students on how to fill in the questionnaire. In addition, the students were assured of their right to withdraw from the study at any time and for any reason. If a student had any query related to the questions, the investigator provided clarification. After the questionnaire was completed, it was handed to the investigator.

### Questionnaire

The questionnaires were newly developed to measure alcohol-related harms based on literature and experts’ opinions. First, the questionnaire was pre-tested among 20 students at Hanoi Medical University to ensure that they clearly understood the meaning of all of the questions.

The questionnaire was divided into two parts.

The first part included questions on demographics (age, gender, type of living situation, and academic year level) and on drinking patterns. For example: ‘How often did you drink at least one full SD of alcohol in the previous 12 months?’ and ‘How often did you drink at least four SDs (for females) or five SDs (for males) per occasion in the previous 12 months’.

Responses were made on a 7-point scale: 0=never (recoded to 0); 1=almost daily (recoded to 7); 2=3–4 days per week (recoded to 3.5); 3=1–2 days per week (recoded to 1.5); 4=1–2 days per month (recoded to 0.375); 5=once per month (recoded to 0.25); and 6=less than once per month (recoded to 0.125). Midpoints of categories were used for the recoding (28, 29). To gain more insight into drinking patterns, questions were also asked about ‘How many SDs did you on average consume per occasion?’ and ‘What is the highest number of SDs you have ever consumed in the previous 12 months?’. These types of answers were coded as the actual numbers of SDs consumed.

The second part of the questionnaire included 23 possible alcohol-related harms, categorized into five main types of harm (see Appendix); these were developed from the literature and based on the opinions of experts. For the present study, a set of items within each of the five types of harm was tested for internal consistency (Cronbach's alpha). The five types of harm are ‘negative influence on daily activities’ (Cronbach's alpha=0.78); ‘social conflict’ (Cronbach's alpha=0.67); ‘loss of control, acute consequences, and withdrawal’ (Cronbach's alpha=0.70); ‘mental health condition and physical illness’ (Cronbach's alpha=0.51); and ‘medical health problems’ (Cronbach's alpha=0.53).

In the first four types of harm, respondents were asked to rate the number of harms experienced during the previous 12 months on a 4-point scale (0=never; 1=one time; 2=two times; and 3=at least three times). For the fifth type (‘medical health problems’), respondents were asked whether they had experienced these harms (0=no and 1=yes) during the previous 12 months. The response to each type of harm is the sum of the positive answers to each of the items indicating that type of harm. For the logistic regression, the sum score was computed by recoding each type of harm into two categories: 0=never and 1=yes. In this way, the sum score indicates the variety of different harms experienced by the respondent.

### Measures

For the present study a standard drink (SD)=1 can of beer (330 ml at 5%)=1 glass of wine (140 ml at 12%)=1 shot of spirit (40 ml at 40%)=12.6 g of pure alcohol.

Abstainers are students who reported not to drink at least one full SD of alcohol in the previous 12 months. Drinkers are students who reported to drink at least one full SD of alcohol in the previous 12 months. Binge drinkers are students who reported to drink at least 4 SDs (for females) or 5 SDs (for males) per occasion in the previous 12 months.

### Data analysis

Analyses were performed with SPSS for Windows (version 15) and STATA (version 10). Cronbach's alpha was calculated to establish the internal consistency of the scales. Descriptive statistics were used to detect differences between male and female students. All other analyses were performed for males and females separately.

Descriptive statistics were used to estimate the frequency and prevalence of alcohol-related harm. Intercorrelations between potential predictors in the multivariate analysis were low (*r*<0.3), except those between the ‘maximum number of SD consumed’ and the ‘average number of SD consumed’ (*r*=0.53). Logistic regression was used to compare the drinkers without alcohol-related harm and those with at least one type of harm. Independent variables were entered in two steps: 1) age and type of living situation; and 2) drinking pattern variables. This allowed us to assess the predictive ability of the drinking pattern variables while controlling for the effects of variables in step 1. In turn, the dependent variables representing the five types of harms were entered separately into the model. Results were presented as odds ratios (OR) with 95% confidence intervals (95% CI).

Poisson regression analyses were then conducted to investigate relationships between age, type of living situation, drinking pattern variables, and number of harms. The number of harms is calculated by summing up how many of the five types of harms the students scored positively (score 0–5). In all multivariate analyses, unweighted data were used.

## Results

The sample population (*N=*1216) included a similar number of female (*n=*606; mean age 20.8 years) and male students (*n=*610; mean age 20.6 years). Regarding living situation, more male than female students lived in a rented house, whereas more female than male students lived in a dormitory or with a family. Male students were twice as likely to be drinkers than female students (Table 1).

**Table 1:** Socio-demographic and drinking behavior characteristics of 1216 medical students in the survey at Hanoi Medical University

Variables	Females (*n*=606)	Males (*n*=610)	*p*	Total (*N*=1216)
Age in years: mean (range)	20.8 (17–26)	20.6 (18–28)	>0.05	20.7 (17–28)
Academic year (%)				
First year	13.5	20.7	<0.001	17.1
Second year	14.5	19.0		16.8
Third year	17.2	16.1		16.5
Fourth year	18.0	15.1		16.5
Fifth year	19.0	13.9		16.4
Sixth year	17.8	15.2		16.5
Type of living situation (%)				
Dormitory	41.7	30.5	<0.001	36.0
Rented house	24.0	43.2		33.6
With family	34.3	26.4		30.3
Drinking behavior (%)				
No	62.3	22.8	<0.001	42.5
Yes	37.7	77.2		57.5

Sample size varies slightly for each category because of missing values.

### Occurrence of harms

Among drinkers, during the previous 12 months male students were significantly more likely to experience harm (81.8%) than female students (60.4%). For male students, the median number of experienced harms was 2, compared with 1 in female students (Table 2).

**Table 2:** Drinking patterns and number of harms by gender in a sample of 699 drinkers in the survey at Hanoi Medical University

Variables	Females (*n*=227)	Males (*n*=466)		Total (*N*=699)
		
	Median [IQR]	Median [IQR]	*p*	Median [IQR]
1. Drinking pattern				
1.1. Frequency of drinking	0.125 [0.125; 0.125]	0.25 [0.125; 0.375]	<0.001	0.125 [0.125; 0.25]
1.2. Frequency of binge drinking	0 [0; 0]	0 [0; 0.125]	<0.001	0 [0; 0.125]
1.3. Average number of standard drinks per occasion	1 [1; 2]	3 [1; 5]	<0.001	2 [1; 3]
1.4. Maximum number of standard drinks consumed	2 [1; 3]	4 [2; 8]	<0.001	3 [2; 6]
2. Occurrence of number of harms				
2.1. Number of types of harms	1 [0; 2]	2 [1; 3]	<0.01	1 [0; 2.5]
2.2. Frequency of number of harms (%)				
0 harm	39.6	18.2	<0.001	25.1
1 harm	27.8	24.1		25.4
2 harms	21.6	26.0		24.5
3 harms	7.5	21.5		16.9
4 harms	2.2	8.8		6.6
5 harms	1.3	1.5		1.4

Sample size varies slightly for each category because of missing values.

The most common type of harm among men and women were ‘loss of control, acute consequences, and withdrawal’ and ‘negative influence on daily activities’. The least common harm among men and women was ‘social conflict’ (Table 3).

**Table 3:** Prevalence of type of harm by gender in a sample of 699 drinkers in the survey at Hanoi Medical University

Variables	Females (*n*=227)	Males (*n*=466)	*p*	Total (*N*=699)
Negative influence on daily activities* (%)	29.4	55.8	<0.001	47.1
Social conflict* (%)	3.1	11.7	<0.001	8.9
Loss of control, acute consequences, withdrawal* (%)	51.8	75.6	<0.001	67.8
Mental health condition and physical illness* (%)	11.9	26.6	<0.001	21.8
Medical health problems* (%)	10.1	13.4	>0.05	12.3

Sample size varies slightly for each category because of missing values.

* Percentage of students scoring 1 or more of these items.

### Association between specific factors and alcohol-related harm

Among female drinkers, very few significant relations were found (Table 4). Only the frequency of drinking per week and age were predictors of a mental health condition and physical illness. Female students who drank more frequently per week were about six times more likely to have experienced mental health conditions and physical problems. The older female students experienced mental health conditions and physical problems less often (Table 4).

**Table 4:** Alcohol-related harm among 228 female drinkers at Hanoi Medical University by socio-demographics and drinking behavior

Model	Negative influence on daily activities		Social conflict		Loss of control		Mental health condition and physical problem		Medical health problems	
				
	OR	95% CI	OR	95% CI	OR	95% CI	OR	95% CI	OR	95% CI
*Model 1*										
Age	0.9	0.8–1.1	0.6	0.4–1.0	1.0	0.9–1.2	0.7**	0.6–0.9	0.9	0.7–1.1
Living situation										
Dormitory vs. with family	1.3	0.7–2.6	na	na	1.0	0.5–1.7	1.5	0.5–4.1	2.1	0.7–6.3
Rent house vs. with family	1.0	0.5–2.3	na	na	1.1	0.5–2.3	1.0	0.3–3.8	1.1	0.3–4.6
*Model 2*										
Age	0.9	0.8–1.1	0.7*	0.5–0.9	1.0	0.5–2.4	0.7***	0.5–0.8	0.9	0.7–1.1
Living situation										
Dormitory vs. with family	1.4	0.7–2.9	na	na	1.0	0.5–1.2	2.0	0.6–6.8	1.7	0.5–5.7
Rented house vs. with family	1.0	0.4–2.3	na	na	1.0	0.5–1.9	1.2	0.3–4.8	0.8	0.2–3.5
Frequency of drinking per week	0.7	0.1–3.2	na	na	1.1	0.5–2.5	6.2*	1.2–33.6	na	na
Frequency of binge drinking per week	1.9	0.9–4.1	na	na	0.0	0.0–3.4	0.6	0.2–1.4	na	na
Maximum number of SD consumed	1.1	1.0–1.3	1.1	0.9–1.6	1.1	1.0–1.3	1.1	1.0–1.3	1.1	0.9–1.5
Average number of SD consumed per occasion	1.1	1.0–1.3	0.4	0.0–2.6	1.3	0.9–1.9	1.0	0.9–1.3	0.9	0.7–1.2

OR=odds ratio; CI=confidence interval; SD=standard drinks;

* *p*<0.05;

** *p*<0.01;

*** *p*<0.001.

Among male drinkers, far more significant relations were found. Predictors of different types of harms are the average number of SD consumed per occasion, age, and type of living situation. Among drinking variables, only ‘average number of SD consumed per occasion’ was associated with a negative influence on ‘daily activities’ and ‘medical health problems’. Male students living in a dormitory or in a rented house were more likely to have experienced a ‘negative influence on daily activities’, ‘mental health condition’, and ‘physical problems’ than those living with a family. Only those living in a dormitory were more likely to experience ‘loss of control, acute consequences, and withdrawal’. The older the male students, the more likely they were to have experienced a ‘negative influence on daily activities’, ‘social conflict’, ‘loss of control, acute consequence, and withdrawal’, ‘mental health condition’, and ‘physical/medical problems’ (Table 5). These findings suggest that the relations between socio-demographic variables and harms were not explained by the drinking pattern variables.

**Table 5:** Alcohol-related harm among 470 male drinkers at Hanoi Medical University by socio-demographic and drinking behavior

Model	Negative influence on daily activities		Social conflict		Loss of control		Mental health condition and physical problem		Medical health problems	
				
	OR	95% CI	OR	95% CI	OR	95% CI	OR	95% CI	OR	95% CI
*Model 1*										
Age	1.20***	1.1–1.3	1.2**	1.1–1.4	1.2*	1.0–1.3	1.1*	1.0–1.3	1.1	1.0–1.3
Living situation										
Dormitory vs. with family	2.2**	1.3–3.7	1.2	0.6–2.7	2.4**	1.4–4.4	2.0*	1.1–3.6	0.9	0.4–1.9
Rented house vs. with family	1.9*	1.1–3.0	1.0	0.5–2.2	1.8*	1.0–3.0	1.9*	1.1–3.5	1.6	0.8–3.2
*Model 2*										
Age	1.2**	1.0–1.3	1.2**	1.1–1.4	1.1*	1.0–1.3	1.1*	1.0–1.2	1.1	1.0–1.3
Living situation										
Dormitory vs. with family	2.1**	1.2–3.8	1.3	0.4–1.5	2.3**	1.3–4.2	2.0*	1.0–3.6	0.9	0.4–1.9
Rented house vs. with family	1.8*	1.1–3.0	1.1	0.3–1.7	1.7*	1.0–3.0	2.0*	1.1–3.6	1.6	0.8–3.1
Frequency of drinking per week	1.0	0.7–1.4	1.3	1.0–1.8	1.0	0.6–1.7	1.2	0.9–1.5	1.1	0.8– 1.6
Frequency of binge drinking per week	4.3	0.1–248.1	1.3	0.9–1.7	2.1	0.1–29.4	1.2	0.9–1.8	1.1	0.8–1.6
Maximum number of SD consumed	1.0	0.9–1.1	1.0	1.0–1.0	1.0	0.9–1.1	1.0	1.0–1.1	1.0	1.0–1.1
Average number of SD consumed per occasion	1.1*	1.1–1.2	1.0	1.0–1.1	1.1	0.9–1.3	1.0	1.0–1.1	1.1	1.0–1.1

OR=odds ratio; CI=confidence interval; SD=standard drinks;

* *p*<0.05;

** *p*<0.01;

*** *p*<0.001.

### Association between specific factors and number of harms

All drinkers who did not experience any harm and experienced at least one harm were included in the analysis. Table 6 shows that age was a significant predictor of the number of harms among female drinkers while age, living away from home, and average number of standard drinks per occasion are significant predictors of the number of harms among male drinkers.

**Table 6:** Poisson regression model for number of harms among 228 female and 470 male drinkers at Hanoi Medical University by socio-demographic and drinking behavior

Model	Female students		Male students	
	
	Coef	95% CI	Coef	95% CI
*Model 1*				
Age	−0.074*	−0.15– − 0.01	0.07***	0.03–0.10
Living situation				
Dormitory vs. with family	0.16	−0.14–0.46	0.27**	0.08–0.45
Rented house vs. with family	0.09	−0.25–0.45	0.23	0.05–0.41
*Model 2*				
Age	−0.09*	−0.02– − 0.02	0.06**	0.03–0.09
Living situation				
Dormitory vs. with family	0.21	−0.11–0.52	0.25**	0.06–0.44
Rented house vs. with family	0.04	−0.33–0.41	0.23*	0.05–0.41
Frequency of drinking per week	0.16	−0.05–0.82	0.06	−0.04–0.15
Frequency of binge drinking per week	0.02	−0.19–0.23	0.08	0.01–0.17
Maximum number of SD consumed	0.04	−0.00–0.09	0.01	−0.00–0.02
Average number of SD consumed per occasion	0.05*	−0.01–0.11	0.02*	0.01–0.03

CI=confidence interval; SD=standard drinks;

* *p*<0.05;

** *p*<0.01;

*** *p*<0.001.

## Discussion

Our findings suggest that alcohol-related harms are common among medical students in Vietnam. The results indicate that female and male students have a similar experience regarding the most common types of harm (‘loss of control, acute consequences, and withdrawal’ and ‘negative influence on daily activities’) and less common types of harm (‘social conflict’) but differ in the prevalence of alcohol-related harms and factors (age, type of living situation, and drinking pattern) that influence harms. Medical students may not differ strongly in terms of drinking from students at other universities in Vietnam. Also, the influence of age and living situation on alcohol-related harm is probably similar for students from other universities.

Our findings are similar to those from other studies worldwide; for example, in New Zealand, the most common negative consequences of alcohol among students are hangover (55%), blackouts (33%), and vomiting (21%) (4), and among students in Australia being sick (12.8%), hangovers (12.3%), and being unable to remember what happened after drinking (10.4%) (21). A study among adolescents in Thailand also indicated that the negative consequences were nausea and vomiting (46.9%), being criticized by someone (38.8%), hangover (37.8%), driving a car or motorcycle after drinking (35.4%), and missing class (32.8%) (13).

In the present study, the prevalence of harm is higher compared with other studies. However, it is difficult to compare the prevalence of alcohol-related harm between studies due to the different measures used. For example, in many studies, each type of harm generally includes only one item, whereas in our study each type of harm included 2–7 items of harm. This means that students in the present study who experienced 1 out of 2–7 items of harm were considered to have alcohol-related harm, leading to a higher prevalence of harm. A Swedish study used a similar measure of collecting data over 12 months and categorized harms into five types (10). This resulted in a prevalence of 43% of them having at least one harm, which is lower than that in Vietnam. Moreover, compared with our students, the prevalence of each type of harm in the Swedish study was also lower, that is, 1) physical health (25.1%); 2) financial situation (12.9%); 3) study or work life (5.5%); 4) family life, marriage, or relationship (1.5%); and 5) friendships or social life (0.9%) (10). The higher negative consequences in our study might be explained by the strength of the alcohol beverages. Medical students in Vietnam who reported drinking home-brewed wine (≥30 proof) or traditional medicinal wine were more likely to develop alcohol problems (5). Other reasons such as coping style, motives and expectancies of drinking, drinking context, and/or drinking location are associated with alcohol-related harms (30) and should be included in future research.

Our results generally confirm that men and women differ in factors that influence alcohol-related harm. In both male and female students, age is a predictor of specific types of harm and of the number of harms. For male students, the higher the age the more harm they experienced. This finding is similar to a study in the United States, showing that being a male student and being older is more likely to lead to alcohol-impaired driving (31). In contrast to men, the older the female student the less harm they experienced. An explanation might be that older female students experienced harm when they were younger and, therefore, they try to minimize such harm when they are older. A study among psychology students in the United States reported that young women were more influenced in their future drinking decisions by their most negative experiences (6).

Type of living situation was the strongest predictor of specific types of harms among male students only. Living with a family seems to be a protective factor, perhaps due to parental control. Our finding is supported by a study among Australian students in which unsupervised drinkers were almost seven times more likely to experience alcohol-related harm than supervised drinkers (21). A study among adolescents in the United States showed that teenagers who are not allowed to drink in school by their parents drink less alcohol during weekends, have a lower frequency of drinking, and tend to experience fewer negative consequences than those who are allowed to drink (20). Another study showed that living in a residence hall led to more opportunities for social activities (such as parties and drinking games), which can lead to alcohol-related harm (32). Thus, intervention programs should take this into account and focus on male students who do not live with a family.

The present study also shows a considerable gender difference in drinking behavior. Male students drank more frequently, engaged in more binge drink, and experienced more alcohol-related harms than female students; this finding is consistent with other studies among students in the United States and Australia (9, 21). The general finding from different countries is that men drink more frequently and in larger quantities per occasion than women, which causes more harm, especially in low-income countries (2). However, in the present study, the gender difference in drinking behavior seems greater than that in other studies, but is consistent with an earlier study among medical students in Vietnam (5). Consistent with other studies, the reported average number of SDs consumed per occasion by men was predictive of specific types of harm. Among women, the reported frequency of drinking was a predictor of their mental health condition and physical problems; this was not found in other studies. It should be noted that the 95% CI (1.2–33.6) is very large for this variable, probably due to the relatively small number of women. This result should be replicated before considering the frequency of drinking to be a definite risk factor for alcohol-related ‘mental health condition’ and ‘physical problems’ among women.

## Study limitations

The present study has several limitations. Participants were students drawn from a single university. Future studies should include more socio-demographic and ethnically diverse samples.

Second, the study design was cross-sectional which allows us to examine the association between drinking and related harm, but not the causal links. Third, because answers to the questionnaires were self-reported, this might entail some level of self-report bias and/or incorrect recall of information.

## Appendix

Alcohol-related harm questions.

During previous 12 months, how often have you experienced the 23 following harms after drinking?

(Relative frequency for each of the 23 harms was rated on a 0 to 3 scale: 0=never, 1=one time, 2=two times, 3=at least three times).

**Table:**

*Negative influence on daily activities*					
1	Negative influence on academic study/miss study/miss night duty in hospital	0	1	2	3
2	Negative influence on housework/activities	0	1	2	3
3	Lost interest in daily activities	0	1	2	3
4	Cannot concentrate on doing anything as normal	0	1	2	3
5	Getting sick then having to stop daily activities	0	1	2	3
6	Financial problems	0	1	2	3
*Social conflict*					
7	Negative influence on family member/flat mate	0	1	2	3
8	Negative influence on friend/colleagues	0	1	2	3
9	Lost close friends/lovers	0	1	2	3
10	Accidents/traffic accident/problems	0	1	2	3
11	Fighting with somebody	0	1	2	3
*Loss of control, acute consequences, withdrawal*					
12	Cannot stop once you have started drinking	0	1	2	3
13	Be drunk and passed out	0	1	2	3
14	Have headache/dizziness/nausea	0	1	2	3
15	Unclear speaking/unsteady step	0	1	2	3
16	Cannot remember what happened when you were drinking	0	1	2	3
17	Feeling guilty or remorseful because of drinking	0	1	2	3
18	Try to cut down on alcohol drinking	0	1	2	3
*Mental health condition and physical illness*					
19	Feeling that mental and physical health is reduced	0	1	2	3
20	Having a memory problem even when one does not drink	0	1	2	3
*Medical health problems*					
21	Problems with physical numbness	0	1	2	3
22	Liver problems	0	1	2	3
23	Stomach problems	0	1	2	3
